# Doping im Schachsport

**DOI:** 10.1007/s00103-026-04251-5

**Published:** 2026-06-11

**Authors:** Thomas E. Wessendorf

**Affiliations:** Ruhrlandklinik, Universitätsmedizin Essen, Universität Duisburg-Essen, Tüschener Weg 40, 45239 Essen, Deutschland

**Keywords:** Schach, Doping, Hirndoping, Stimulanzien, Cheating, Chess, Doping, Neuro enhancement, Stimulants, Cheating

## Abstract

Der Gebrauch leistungssteigernder Substanzen ist auch im Schachsport möglich: Für Stimulanzien zum kognitiven Doping wie Amphetamine, Methylphenidat, Modafinil und Ephedrin gibt es Hinweise auf eine gewisse Leistungssteigerung, allerdings auch auf negative Effekte im Hinblick auf Entscheidungsprozesse unter Zeitdruck. Die Substanzen stellen allgemein ein Gesundheitsrisiko dar; insbesondere bei Überdosierungen können sie das Herz-Kreislauf-System belasten oder psychotische Symptome auslösen. Grundsätzlich besteht zudem das Risiko einer Abhängigkeit oder einer Funktion als „Einstiegsdroge“.

Als anerkannte Sportart werden auch im organisierten Schach Dopingkontrollen durchgeführt. Diese sind jedoch auf den Spitzensport beschränkt und entsprechend selten, weshalb bislang keine relevanten Dopingfälle bekannt sind. Demgegenüber wird im Leistungs- und Amateursport eine höhere Prävalenz sowohl bei Profis als auch bei Amateuren angenommen. Die größere Gefahr für das Fairplay im Schach liegt jedoch in anderen betrügerischen Methoden (Cheating, elektronisches Doping).

## Abstract (en)

The use of performance-enhancing substances is also possible in chess. Regarding stimulants used for cognitive doping—such as amphetamines, methylphenidate, modafinil, and ephedrine—there is evidence suggesting a slight improvement in performance; however, there is also evidence of negative effects on decision-making processes under time pressure. These substances generally pose a health risk; particularly in cases of overdose, they can strain the cardiovascular system or trigger psychotic symptoms. Furthermore, there is a fundamental risk of addiction or of the substance serving as a “gateway drug.”

As chess is recognized as an official sport, doping controls are conducted within organized competitions. However, these are limited to elite-level competition and are consequently rare; as a result, few significant doping cases have come to light thus far. In contrast, a higher prevalence is suspected in competitive and amateur chess—among both professionals and amateurs alike. Nevertheless, the greater threat to fair play in chess lies in other fraudulent methods (such as cheating or electronic doping).

## Einleitung

Im Nationalen Anti-Doping Code (NADC) der Nationalen Anti Doping Agentur Deutschland (NADA) wird Doping definiert als das Vorliegen eines oder mehrerer in Artikel 2 festgelegten Verstöße gegen Anti-Doping-Bestimmungen [1]. Im engeren Sinne wird unter dem Begriff Doping der Gebrauch von verbotenen Substanzen verstanden, der durch den Nachweis dieser Substanz belegt wird. Doping betrifft neben den bekannten olympischen Disziplinen auch nichtolympische Sportarten wie Schach: Der Deutsche Schachbund (DSB) als Mitglied im Weltschachbund (Fédération Internationale des Échecs, FIDE) muss den Forderungen des International Olympischen Komitees (IOC) und dem Welt-Anti-Doping-Code (WADC) nachkommen. Der DSB hat sich „sauberen Sport“ auf die Fahnen geschrieben, engagiert sich in der Anti-Doping-Arbeit und Dopingprävention gerade bei Jugendlichen, auch als Teil des Netzwerks „Gemeinsam gegen Doping“ zusammen mit der NADA [2].

In diesem Artikel werden zunächst die Substanzen, die im Schachsport zum kognitiven Doping eingesetzt werden könnten, sowie deren Wirkungen und Risiken kurz dargestellt. Es folgt eine Beschreibung der wenigen relevanten Dopingfälle sowie ein Überblick über wissenschaftliche Forschung zur Prävalenz. Anschließend wird das elektronische Doping (E-Doping) erläutert, das im Vergleich zum pharmakologischen Doping eine deutlich größere Bedeutung im Schachsport hat.

## Eingesetzte Substanzen

Das Schachspiel gilt als „Probierstein des Gehirns“ [3]. Bei einer klassischen Partie am Brett („over the board“, OTB) unter üblichen Wettkampfbedingungen mit einer Dauer von 4–5 h werden von den Sportlern in erster Linie ausdauernde Konzentrationsfähigkeit, Aufmerksamkeit und Wachheit verlangt. Dies gilt auch beim Online-Schach, bei dem zumeist deutlich kürzere Bedenkzeiten gewählt werden (Partiedauer im Blitzschach 5–10 min, im Bulletschach 2–3 min), da viele Partien häufig hintereinander über mehrere Stunden gespielt werden.

Zur Leistungssteigerung im Schachsport kommen daher in erster Linie zentral stimulierende Substanzen in Betracht: Die Einnahme verbotener oder verschreibungspflichtiger Substanzen wie Amphetamine, Ephedrin, Methylephedrin, Pseudoephedrin, Methylphenidat oder Modafinil steigert bei Gesunden den Wachheitsgrad und die Daueraufmerksamkeit, wobei Amphetamine die stärksten Effekte zeigen, gefolgt von Modafinil und Methylphenidat. In einzelnen Studien gezeigte Verbesserungen des Gedächtnisses sind mit hoher Wahrscheinlichkeit sekundär als Folge der gesteigerten Konzentration und Aufmerksamkeit zu interpretieren. Alle Effekte sind bei Müdigkeit (z. B. infolge von Schlafentzug) stärker ausgeprägt. Koffein – als legales Stimulans bei Gesunden – zeigt in höheren Dosen vergleichbar starke Effekte [4].

Hierzu gibt es wissenschaftliche Untersuchungen auch im Schachsport: Im Rahmen einer randomisierten, placebokontrollierten, doppelblinden Studie von Franke et al. aus Mainz [5] spielten 39 Schachspieler insgesamt über 3000 Partien gegen ein kommerzielles Schachprogramm (Fritz 12), angepasst an ihre Spielstärke, jeweils nach der Einnahme von Modafinil, Methylphenidat, Koffein oder Placebo. Die Teilnehmer konnten nach der Einnahme der Substanzen mehr Partien gegen den Computer gewinnen als nach der Einnahme von Placebo (je nach Stimulans 3,1–5,9 %). Der Effekt erscheint vergleichsweise gering, könnte aber in einem Turnier über 9 Runden einen halben Punkt mehr bedeuten. Allerdings wurde dieser Effekt teilweise dadurch aufgehoben, dass mehr Bedenkzeit in Anspruch genommen wurde, sodass unter Einfluss der Substanzen mehr Partien auf Zeit verloren wurden. Zusätzliche neuropsychologische Testungen zeigten zudem, dass Entscheidungsprozesse unter Zeitdruck – und solche werden im Turnierschach verlangt – durch psychoaktive Substanzen auch negativ beeinflusst werden können.

Diese Medikamente stellen prinzipiell ein Gesundheitsrisiko für die Sportler dar. Die am häufigsten eingesetzten Stimulanzien Amphetamine und Methylphenidat blockieren präsynaptische Noradrenalin- und Dopamintransporter und steigern die monoaminerge Neurotransmission. Amphetamine stimulieren zusätzlich die Dopaminfreisetzung [4]. Durch die sympathomimetischen Effekte lassen sich die Nebenwirkungen insbesondere bei Überdosierungen erklären, auch wenn die beim Hirndoping eingesetzten Dosen in der Regel den therapeutischen Dosierungen entsprechen. Typische Nebenwirkungen sind beschleunigter Herzschlag, Bluthochdruck oder Schlaflosigkeit. In sehr seltenen Fällen können Agitiertheit bis hin zu psychotischen Veränderungen auftreten [4]. In einer Fall-Kontroll-Studie konnte ein Zusammenhang zwischen dem Gebrauch von Stimulanzien und einem plötzlichen unerklärten Herztod gefunden werden [6].

Grundsätzlich besteht ein Abhängigkeitspotenzial. Das Suchtrisiko und die Gefahr von Stimulanzien, als „Einstiegsdroge“ zu wirken, werden jedoch kontrovers diskutiert: Beispielsweise wird bei der Therapie einer Aufmerksamkeitsdefizit‑/Hyperaktivitätsstörung (ADHS) einerseits die Gefahr eines Substanzmissbrauchs reduziert, andererseits ist der Einsatz von Stimulanzien als Erstlinientherapie mit Medikamentenmissbrauch assoziiert [7].

## Bisher bekannte Dopingfälle

Bisher konnten die genannten Substanzen bei den Athleten allerdings noch nicht nachgewiesen werden. Die bisher einzige positive Dopingprobe bei Deutschen Meisterschaften gab es im Jahr 2017, als bei einem Teilnehmer die Substanz Hydrochlorothiazid nachgewiesen wurde. Dieses harntreibende Mittel steht auf der Verbotsliste, da es die Einnahme leistungssteigernder Substanzen maskieren kann. Dem Athleten war nicht bewusst, dass er zu diesem Zeitpunkt eigentlich eine medizinische Ausnahmegenehmigung gebraucht hätte; die Einnahme des Mittels erfolgte als blutdrucksenkendes Medikament, was nachträglich durch ein fachärztliches Attest bestätigt werden konnte, sodass letztlich der positive Test folgenlos blieb. Grundsätzlich muss als Einschränkung hervorgehoben werden, dass sich bisher die durch die NADA durchgeführten Dopingkontrollen im Schach nur auf deutsche Einzelmeisterschaften beschränken. So wurden auf dem Schachgipfel 2022 in Magdeburg in 3 Turnieren insgesamt 9 Urinproben genommen. Zum Vergleich: Die 429 deutschen Olympia-Teilnehmenden in Paris wurden allein im Vorfeld der Spiele 1176-mal kontrolliert.

Der fehlende Nachweis von Substanzen liegt nicht in einer zu geringen Sensitivität begründet, da bei den Urinproben, der einzigen Dopingkontrollmethode im Schach, beispielsweise Amphetamine 1 bis 3 Tage nach dem Konsum nachweisbar sind [8]. Im Gegensatz zu körperlichen Sportarten, bei denen beispielsweise Medikamente zum Muskelaufbau in Trainingsphasen außerhalb von Wettkämpfen eingesetzt werden könnten, macht der Einsatz von Stimulanzien im Schachsport aufgrund des Wirkprinzips prinzipiell nur im Wettkampf, z. B. während eines Turniers, Sinn.

Unter Schachspielern selbst sind die Dopingkontrollen durchaus umstritten:

Der langjährige Vorkämpfer des Deutschen Schachs, der inzwischen verstorbene Großmeister Dr. Robert Hübner, lehnte nach der Einführung von Dopingkontrollen im internationalen Schach im Jahr 2000 weitere Einsätze in der Nationalmannschaft ab [9]. Seiner Ansicht nach existierte im Schach keine sinnvolle medikamentöse Leistungssteigerung.

Der ukrainische Großmeister Wassili Iwantschuk, damaliger Weltranglisten-Dritter, geriet in Verdacht, bei der Schach-Olympiade 2008 in Dresden (entspricht der Mannschafts-WM im Schach) den Dopingtest verweigert zu haben, was einem positiven Testergebnis entspricht. Tatsächlich hatte er nach einer misslichen Niederlage in der letzten Runde, die den undankbaren 4. Platz bedeutete, die Kontrolleure aus Frust und Enttäuschung unbeabsichtigt stehen gelassen. Letztlich blieb dies sowohl für die Mannschaft als auch für den Spieler ohne Folgen, nachdem ursprünglich noch eine 2‑jährige Sperre im Raum gestanden hatte.

Andere „Dopingfälle“ bzw. positive Dopingproben im Weltschach sind dem Autor nicht bekannt.

Ein tragischer Fall machte aber aktuell Schlagzeilen: Der US-amerikanische Großmeister Daniel Naroditzky wurde im Oktober 2025 im Alter von 29 Jahren tot aufgefunden: Nach den veröffentlichten Ergebnissen der Autopsie war die Todesursache vermutlich eine Herzrhythmusstörung im Rahmen einer kardialen Sarkoidose. Die toxikologische Untersuchung wies allerdings zum Todeszeitpunkt Methamphetamin, Amphetamin, Mitragynin und 7‑Hydroxymitragynin in seinem Körper nach. Die Konzentrationen der Substanzen lagen jedoch nicht im toxischen Bereich, sodass es letztlich keinen Hinweis für eine beabsichtigte oder unbeabsichtigte Überdosierung gab. Der Tod wurde daher als Unfall eingestuft. Allerdings wurde auf das potenzielle kardiovaskuläre Risiko der Stimulanzien (s. oben) hingewiesen. Ob die Sarkoidose zu Lebzeiten von Naroditzky diagnostiziert worden war oder ob die nachgewiesenen Substanzen legal verschriebene Medikamente waren, ist nicht publiziert [10].

## Prävalenz des Dopings im Schach

Die negativen Ergebnisse der Dopingproben stehen in gewisser Weise im Gegensatz zu wissenschaftlichen Erhebungen zur Häufigkeit von Doping im Leistungs- oder Amateursport sowie zur generellen Bereitschaft gesunder Menschen, Neuroenhancement („Hirndoping“) zu betreiben: Bei einer webbasierten Umfrage unter 4580 amerikanischen College-Studenten durch Teter et al. zeigte sich bereits 2006 eine Lebenszeitprävalenz von 8,3 % für die Anwendung illegaler Stimulanzien, in erster Linie oraler Amphetamine sowie Methylphenidat. Als Hauptmotive wurden eine gesteigerte Konzentrationsfähigkeit und erhöhte Wachheit angegeben [11].

Vergleichbare Ergebnisse konnten an der Universität Zürich erhoben werden: Ott und Biller-Andorno verglichen mittels Online-Fragebogen bei 1765 Studenten diejenigen mit Konsum von Stimulanzien gegenüber denjenigen ohne: Insgesamt 4,7 % hatten diese Substanzen (in erster Linie Methylphenidat, aber auch Amphetamin und Modafinil) für das Studium eingesetzt. Diese Studierenden waren eher männlich, weniger religiös und hatten mehr Erfahrungen mit Drogen [12].

In Examenssituationen berichten Dietz und Striegel über die Anwendung verschreibungspflichtiger Medikamente bei Studierenden in Deutschland: Mittels schriftlichen Fragebogens und der Randomized-Response-Technik (RTT) wurden 2569 Studenten befragt. Die Prävalenz war höher bei Männern (23,7 % vs. 17 %), am höchsten in Sport-assoziierten Studiengängen und in jüngeren Semestern. Die geschätzte 12-Monats-Prävalenz betrug insgesamt 20 % [13].

Franke et al. führten bei über 7000 erwachsenen Schachspielern eine schriftliche bzw. Online-Umfrage zum Einsatz legaler oder illegaler Substanzen zur Verbesserung der Spielstärke durch: Mittels RTT schätzten die Autoren bei einer Antwortrate von insgesamt 38 %, dass 25 % der Schachspieler frei verfügbare Substanzen und 8,9 % verschreibungspflichtige oder illegale Mittel zur Leistungsverbesserung eingesetzt hatten (Lebenszeitprävalenz). Interessanterweise lag der Anteil der Letzteren bei anonymen Fragebögen deutlich niedriger (3,5 %). Obwohl diese Zahlen unter den geschätzten Dunkelziffern für Doping in anderen Sportarten liegen, schließen die Autoren, dass der Substanzeinsatz im Schachsport weiterer Aufmerksamkeit bedarf, da neben den gesundheitlichen Risiken und der Gefahr einer Abhängigkeit auch das Prinzip des Fairplay im Sport gefährdet wird [14].

Legales kognitives Enhancement oder illegales kognitives Doping werden nicht nur beim Schach, sondern auch bei anderen Sportarten eingesetzt: Nach Selbstberichten wenden 28 % von Pokerspielern und 23 % von E‑Sportathleten verschreibungspflichtige Medikamente an [15], Koffein als legale Substanz wurde bei 71 % bzw. 67–78 % der Sportler regelmäßig eingesetzt [16].

Unter Verwendung eines anonymen Fragebogens und der RTT schätzten Dietz et al. die Prävalenz des physischen, aber auch des kognitiven Dopings bei knapp 3000 Hobbytriathleten auf 13 % bzw. 15 % [17]. Interessanterweise zeigte sich eine Assoziation zwischen physischem und kognitivem (erlaubtem) Enhancement und (verbotenem) Doping. Die Autoren folgern daraus, dass es Athleten gibt, die eine generelle Neigung zu leistungssteigernden Substanzen haben.

In einer aktuellen Online-Umfrage unter 1924 deutschen Vereinsschachspielern von Schu und Haller ergab sich eine Prävalenz für das Jahr 2023 von 5,1 % für kognitives Doping [18]. Die Prävalenz war etwas geringer bei schwächeren und älteren Spielern, war aber für klassisches Schach am Brett oder Online-Schach vergleichbar. Spitzenspieler, die sich bei Deutschen Meisterschaften Kontrollen unterziehen müssten, waren in dieser Umfrage allerdings vergleichsweise gering vertreten.

## E-Doping

Wenn man die Ergebnisse der Studie von Schuh und Haller auf ein typisches Turnierergebnis extrapoliert (Steigerung der erzielten Punkte, siehe oben), ist der Effekt allerdings vernachlässigbar klein im Vergleich zu Betrugsmöglichkeiten gerade im Schach:

Der Einsatz elektronischer Hilfsmittel ermöglicht es Schachspielern jeglicher Spielstärke, die besten Großmeister zu schlagen. Die aktuell führenden Schachprogramme werden auf eine Spielstärke von ca. 3400 ELO^1^ geschätzt (zum Vergleich der derzeit beste Spieler Magnus Carlsen mit 2840) und können leicht auf einem Smartphone eingesetzt werden. Der Einsatz eines solchen in wichtigen Phasen einer Partie, zum Beispiel getarnt durch einen Toilettenbesuch, wäre sicher spielentscheidend.

So konnte dem lettischen Großmeister Igor Rausis der Gebrauch eines Smartphones zur Partieanalyse während der Partie nachgewiesen werden, was rückblickend eine sehr ungewöhnliche Leistungssteigerung im Alter von mehr als 50 Jahren mit einem Zuwachs von mehr als 200 ELO-Punkten erklären dürfte. Der Großmeistertitel wurde ihm aberkannt [19].

Es sind zahlreiche weitere solcher Fälle auf Profi-, aber auch Amateurniveau im Internet dokumentiert [20]. Dieser im Gegensatz zum pharmakologischen Doping „Cheating“ oder auch „elektronisches Doping“ (E-Doping) genannte Betrug stellt für den Schachsport auf allen Leistungsniveaus eine eminente Bedrohung dar. Der Deutsche Schachbund unterhält seit Jahren eine Anti-Cheating-Kommission, und Schiedsrichter werden in speziellen Lehrgängen zu diesem Thema geschult.

Turnierveranstalter müssen entsprechende Anti-Cheating-Maßnahmen ergreifen, die an Umfang die derzeit durchgeführten klassischen Dopingkontrollen weit übertreffen. Die Schachsportler werden bei wichtigen Turnieren durch den Einsatz von Metalldetektoren vor der Partie untersucht. Der Einsatz von Mobiltelefonen oder Smartwatches, aber auch anderen elektronischen Geräten ist strikt untersagt. Diese werden während der Partie in speziellen Boxen aufbewahrt. Wenn bei einem Spieler im Spielbereich ein verbotenes elektronisches Gerät gefunden wird, verliert er die Partie.

## Fazit

Durch den Einsatz pharmakologisch wirksamer Substanzen, in erster Linie Stimulanzien, kann im Schachsport möglicherweise eine gewisse Leistungssteigerung erreicht werden. Die Tatsache, dass bei den wenigen offiziellen Dopingtests bisher kaum Fälle erkannt wurden, steht im Widerspruch zu der Bereitschaft und Häufigkeit von allgemeinem „Hirndoping“ in der Gesellschaft. Gesundheitliche Schäden durch pharmakologisches Doping dürften relevant sein. Im Hinblick auf Fairplay im Schachsport ist E‑Doping eine viel größere Bedrohung.

## Data Availability

Alle dieser Arbeit zugrunde liegenden Daten sind in diesem Artikel enthalten.

## Footnotes

1 ELO leitet sich vom Nachnamen des ungarisch-amerikanischen Physikers und Schachspielers Arpad Elo ab, der das Elo-Rating-System entwickelt hat. Der Wert spiegelt die Spielstärke von Schachspielern wider.
